# Left Main Coronary Stenosis as a Consequence of Bentall Operation: Percutaneous Treatment

**DOI:** 10.4061/2009/213954

**Published:** 2009-10-27

**Authors:** Manrico Balbi, Flavio Scarano, Gian Paolo Bezante

**Affiliations:** Department of Cardionephrology and Department of Internal Medicine, University of Genova, Viale Benedetto XV/6–16132 Genova, Italy

## Abstract

A 65-year-old man suffering from ascending aorta aneurysm and atherosclerotic three vessel disease without left main involvement underwent aortic root replacement with coronary ostia reimplantation according to the modified Bentall technique and multiple coronary artery bypass grafts. Gelatin-resorcin-formaldehyde glue was used to reinforce the aortic coronary buttons and to facilitate hemostasis. Five months after surgery, the patient experienced rapidly worsening effort angina. Coronary angiography showed severe left main narrowing. The considerable amount of time that elapsed between surgery and the onset of symptoms implies that the problem was not related to an imperfect suture technique, but was most likely caused by an inflammatory and proliferative response to the glue that had been used. We performed elective percutaneous coronary intervention and stenting of the protected coronary vessel without technical difficulties and with a satisfactory final result. The patient is currently symptom-free after 1 year's follow-up.

## 1. Introduction

Bentall-type surgery for aortic root repair may be complicated by ostial coronary stenosis, and surgical or percutaneous revascularization may be required.

We report a case of left main coronary stenosis five months after a Bentall operation associated with coronary artery bypass graft (CABG), which was treated by percutaneous coronary intervention (PCI) in order to avoid a redo surgery.

## 2. Case Report

**Figure 1:**
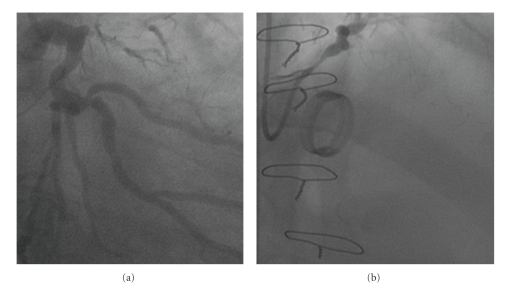
(a) Left coronary arteriogram in the right anterior oblique (RAO) projection before coronary intervention. (b) Left coronary arteriogram in the left anterior oblique (LAO) projection five months after surgery.

A 65-year-old man suffering from ascending aorta aneurism and atherosclerotic three vessel disease without left main coronary (LM) involvement (Figure 1(a)) underwent aortic root replacement with a 27 mm Dacron valve graft conduit. The coronary ostia was reimplanted according to the modified Bentall technique, and coronary artery bypass graft was performed (left internal mammary to left anterior descending, saphenous grafts to marginal and posterolateral branches). Gelatin-resorcin-formaldehyde glue was used to reinforce the aortic coronary buttons and to facilitate hemostasis.

Five months after surgery, the patient experienced rapidly worsening effort angina. Coronary angiography showed severe LM narrowing (Figure 1(b)) and total ostial occlusion of the saphenous graft to marginal branch.

We considered the new LM ostial stenosis together with the graft occlusion to be the culprit lesion, jeopardizing a considerable amount of myocardium in the circumflex territory. In order to avoid the risks connected to a redo surgery or to an attempt to recanalize a completely thrombized venous graft, we decided to treat this “protected” LM stenosis by PCI.

**Figure 2:**
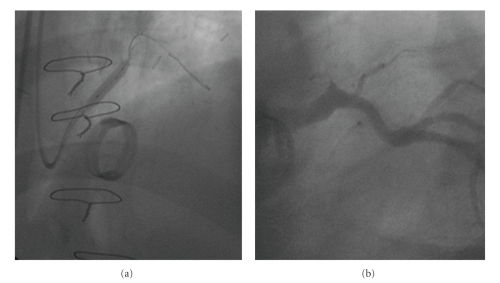
(a) Left coronary arteriogram in the left anterior oblique (LAO) projection during balloon inflation. (b) Left coronary artery in LAO projection after stenting, demonstrating good angiographic result.

A 0.014′′ HT Floppy II guidewire (Abbott Vascular, Diegem, Belgium) was advanced into the marginal branch through a 6 French Amplatz Left guiding catheter (Z2 Medtronic, Minneapolis, MN). Then we predilated the LM with a Maverick balloon 3.5 × 20 mm (Boston Scientific, Natick, MA), in order to avoid an expected underexpansion of a stent into the tough, fibrotic lesion due to the inflammatory response to glue (Figure 2(a)). Finally we deployed a stent Libertè 4.5 × 20 mm. (Boston Scientific, Natick, MA) inflated up to 18 atmospheres for 30 seconds. No technical difficulties occurred, and we obtained a satisfactory final result (Figure 2(b)). The patient had an uneventful course following the procedure and is currently symptom-free after one year's follow-up.

## 3. Discussion

Resorcin-formol-glue is occasionally used to treat hemorrage associated with coronary artery reimplantation during Bentall operation. This may lead to an inflammatory and proliferative response, with subsequent extrinsic compression and narrowing of the coronary ostia [1]. Other causes of coronary stenosis after Bentall operation are an imperfect suture technique or a direct damage of the coronary ostia caused by technical issues including twist, tension, external packing, and trauma due to the instrumentation for direct anterograde cardioplegia.

In this case, the considerable amount of time that elapsed between surgery and the onset of symptoms made us hypothesize that the problem was not related to suture technique or direct damages but was most likely caused by the glue that had been used [2]. The treatment of choice in this particular type of ostial stenosis still remains CABG, and negative results of PCI are reported in literature [3]. However, our patient has already had CABG at the time of aortic repair, but unfortunately five months later he experienced not only the LM narrowing but also the occlusion of the saphenous vein graft to the marginal branch. In complex cases like this CABG redo is a heavy risky option; on the basis of the results we obtained in this case and of our previous experience [4], we believe that PCI may be a reliable alternative.
